# One-Step Photocontrolled Polymerization-Induced Self-Assembly (Photo-PISA) by Using In Situ Bromine-Iodine Transformation Reversible-Deactivation Radical Polymerization

**DOI:** 10.3390/polym12010150

**Published:** 2020-01-07

**Authors:** Haihui Li, Qinghua Xu, Xiang Xu, Lifen Zhang, Zhenping Cheng, Xiulin Zhu

**Affiliations:** State and Local Joint Engineering Laboratory for Novel Functional Polymeric Materials, Jiangsu Key Laboratory of Advanced Functional Polymer Design and Application, Suzhou Key Laboratory of Macromolecular Design and Precision Synthesis, College of Chemistry, Chemical Engineering and Materials Science, Soochow University, 199 Ren-ai Road, Suzhou 215123, China; harvey1119@163.com (H.L.); 15250060196@163.com (Q.X.); xudongqi1818@163.com (X.X.); xlzhu@suda.edu.cn (X.Z.)

**Keywords:** photopolymerization, reversible-deactivation radical polymerization (RDRP), polymerization-induced self-assembly (PISA), bromine-iodine transformation, photocontrolled

## Abstract

Polymerization-induced self-assembly (PISA) has become an effective strategy to synthesize high solid content polymeric nanoparticles with various morphologies in situ. In this work, one-step PISA was achieved by in situ photocontrolled bromine-iodine transformation reversible-deactivation radical polymerization (hereinafter referred to as Photo-BIT-RDRP). The water-soluble macroinitiator precursor α-bromophenylacetate polyethylene glycol monomethyl ether ester (mPEG_1k_-BPA) was synthesized in advance, and then the polymer nanomicelles (mPEG_1k_-*b*-PBnMA and mPEG_1k_-*b*-PHPMA, where BnMA means benzyl methacrylate and HPMA is hydroxypropyl methacrylate) were successfully formed from a PISA process of hydrophobic monomer of BnMA or HPMA under irradiation with blue LED light at room temperature. In addition, the typical living features of the photocontrolled PISA process were confirmed by the linear increase of molecular weights of the resultant amphiphilic block copolymers with monomer conversions and narrow molecular weight distributions (*M*_w_/*M*_n_ < 1.20). Importantly, the photocontrolled PISA process is realized by only one-step method by using in situ photo-BIT-RDRP, which avoids the use of transition metal catalysts in the traditional ATRP system, and simplifies the synthesis steps of nanomicelles. This strategy provides a promising pathway to solve the problem of active chain end (C-I) functionality loss in two-step polymerization of BIT-RDRP.

## 1. Introduction

In the past 20 years, the development of polymer synthesis technology has brought convenience to the preparation of polymers with different topologies, such as linear, grafted, branched, and ring-shaped. Intensive studies have shown that under certain conditions, existing polymers with different structures can self-assemble to form structured aggregates. Among these polymers, the linear block copolymer has the simplest structure, so the investigation of its self-assembly behaviour is the most extensive and in-depth [1,2]. The self-assembly of block copolymers has a wide range of applications in materials, chemistry, nanoscience, and biomedicine [3,4]. However, in the conventional method, in order to obtain a better nanostructure morphology, the concentration of the block copolymer solution needs to be highly diluted [5,6], which limits its potential application. In recent years, polymerization-induced self-assembly (PISA) has attracted great attention [7]. In a PISA, the polymerization and the self-assembly processes are performed simultaneously, so the synthesis steps are greatly simplified compared to the conventional self-assembly method [8,9]. In addition, PISA has many advantages over traditional methods, such as rich assembly morphologies [7], high solid content [10], and suitability for various solvents [11,12], and the morphologies of the nanostructures can be controlled by adjusting the molar ratio of hydrophilic and hydrophobic segments during PISA process [13]. PISA is theoretically suitable for all reversible-deactivation radical polymerization (RDRP) such as atom transfer radical polymerization (ATRP), reversible addition-fragmentation chain transfer (RAFT) polymerization and iodine transfer radical polymerization (ITP) [7,14,15,16,17]. However, the more in-depth study of PISA is currently carried out by RAFT polymerization due to the absence of transition metal during PISA process [7,18,19,20,21].

**Scheme 1:**
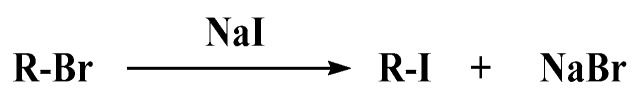
In situ nucleophilic substitution reaction.

Alkyl iodides (R-I) are usually used as initiators and regulators in iodine-mediated RDRP [22,23,24,25]. However, the bond dissociation energy of the C-I bond is relatively low, so alkyl iodides are usually unstable and need special storage conditions, which limits the practical application of this polymerization method [26]. Recently, Goto’s group [26] and our group [27] reported thermal-initiated and photocontrolled in situ bromine-iodine transformation RDRP (BIT-RDRP) strategies, respectively. This method generates R-I in situ in a polymerization system by a nucleophilic substitution reaction of a relatively stable atom transfer radical polymerization (ATRP) initiator alkyl bromide (R-Br) with NaI (Scheme 1). Since commercially available ATRP R-Br initiators are used as the precursors in a BIT-RDRP process, this strategy avoids the introduction of transition metal catalysts usually used in a transition metal-mediated ATRP system. On the other hand, a PISA usually requires a two-step procedure. Generally, the solvent-soluble block is obtained in the first step, and then it is used as a macroinitiator to initiate the polymerization of the nucleating monomer to form an amphiphilic block copolymer in situ [28]. Very recently, our group introduced the first photo-BIT-RDRP into a two-step PISA process, and different micelle morphologies (spherical micelles and vesicle aggregates) were controllably generated by the two-step photo-BIT-RDRP-PISA method using polyethylene glycol methacrylate (PEGMA) and benzyl methacrylate (BnMA) as hydrophilic and hydrophobic monomer, respectively [29]. Through this strategy, we have successfully established a new PISA method by combination the advantages of both ATRP and iodine-mediated RDRP, which avoiding the use of transition metals in the ATRP and overcoming the shortcomings of the unstability of alkyl iodide initiator in the iodine-mediated RDRP method [29].

**Scheme 2:**
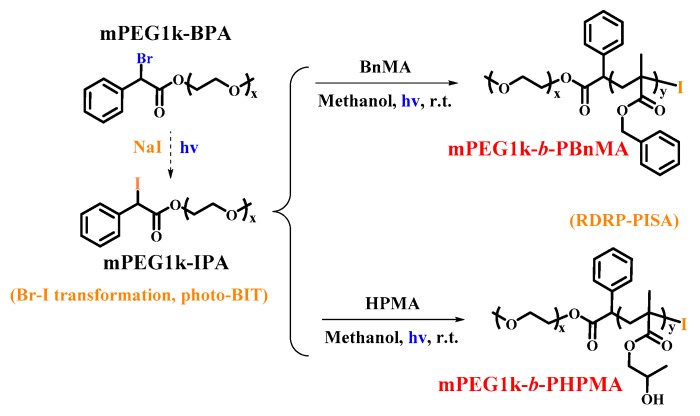
Synthetic routes of mPEG_1k_-*b*-PBnMA and mPEG_1k_-*b*-PHPMA nanoparticles by using one-step photo-BIT-RDRP-PISA strategy.

It is well-known that the functional chain end of the polymer-I (C-I bond) is relatively active and easy to cause an unavoidable functionality loss during the process of conventional polymerization post-treatment. Therefore, there is a drawback of functionality loss of the macroinitiator polymer-I obtained in the first step for the two-step photo-BIT-RDRP-PISA mentioned above. Therefore, it is highly desired to construct a one-step in situ bromine-iodine transformation RDRP-PISA (BIT-RDRP-PISA) system, especially photo-BIT-RDRP-PISA system, which will simplify the polymerization procedure and reduce the loss of active C-I chain ends significantly. Based on this purpose, in this work, we first focused on the synthesis of a water-soluble alkyl bromide as a macroinitiator precursor (mPEG_1k_-BPA) instead of R-Br in the two-step photo-BIT-RDRP-PISA. Subsequently, the one-step photo-BIT-RDRP-PISA process (Scheme 2) was smoothly carried out by using mPEG_1k_-IPA, a product of in situ bromine-iodine transformation nucleophilic substitution of mPEG_1k_-BPA with NaI [27,29], as the alkyl iodide macroinitiator to initiate a PISA process in methanol in the presence of the hydrophobic monomer (BnMA or hydroxypropyl methacrylate (HPMA)) under irradiation with blue LED light at room temperature.

## 2. Experimental

### 2.1. Materials

Benzyl methacrylate (BnMA, 98%, J&K, Shanghai, China) and hydroxypropyl methacrylate (HPMA, 99%, Energy Chemical, Shanghai, China) were passed through an alumina column to remove the inhibitor. Polyethylene glycol monomethyl ether (mPEG_1k_, *M*_n_ = 1000 g/mol, 97%) was purchased from TCI (Shanghai, China). 2-Bromo-2-phenylacetic acid (98%), sodium iodide (97%) and triethylamine (99.5%) were also purchased from TCI (Shanghai, China). Dichlorosulfane (98%), anhydrous methanol (99.5%), anhydrous ether, tetrahydrofuran (99.5%) and all other chemicals were obtained from Shanghai Chemical Reagents Co. Ltd. (Shanghai, China) and used as received unless otherwise mentioned.

### 2.2. Characterization

The number average molecular weight (*M*_n,GPC_) and molecular weight distribution (*M*_w_/*M*_n_) values of the resulting polymers were determined using a HLC-8320 gel permeation chromatograph (GPC, TOSOH, Kyoto, Japan) equipped with a refractive-index detector (TOSOH), using a TSKgel guard column SuperMP-N (4.6 × 20 mm) and two TSKgel SupermultiporeHZ-N (4.6 × 150 mm), with measurable molecular weights ranging from 5 × 10^2^ to 5 × 10^5^ g/mol. THF was used as an eluent at a flow rate of 0.35 mL/min operated at 40 °C. GPC samples were injected using a TOSOH plus autosampler and calibrated with polymethyl methacrylate (PMMA) standards purchased from TOSOH. ^1^H NMR spectra of the polymers were recorded on a Bruker (Beijing, China) 300 MHz nuclear magnetic resonance (NMR) instrument using CDCl_3_ or DMSO-*d*_6_ as the solvent and tetramethylsilane (TMS) as an internal standard at ambient temperature. The UV-visible absorption spectrum was measured by a UV-2600 UV-Vis spectrophotometer (Shimadzu, Kyoto, Japan) with methanol as the solvent. The morphology of the polymeric nanoparticles was obtained by a TecnaiG22 transmission electron microscope (TEM, FEI, Hillsboro, OH, USA) with an accelerating voltage of 120 kV. Take 4 μL of the polymer mixture solution in a dry and clean ampule, dilute with 5 mL of methanol solvent, and then pipet 10 μL of the solution (0.5 mg/mL) onto a 200 mesh carbon-coated copper mesh. After standing for 40 s, filter paper was used to remove excess solvent from under the copper mesh. In order to facilitate the observation of the morphology of the polymer nanoparticles, it is necessary to dye the aqueous solution with a concentration of 1.0% *w/w* of phosphotungstic acid. Therefore, 10 μL of an aqueous solution of phosphotungstic acid was pipetted onto a copper mesh to which polymeric nanoparticles had been dropped. After standing for 20 s, filter paper was used to remove excess solvent from under the copper mesh. Dynamic light scattering (DLS) measurements were conducted using a NanoBrook 90 Plus instrument (BIC, Huntsville, NY, USA). The intensity weighted mean hydrodynamic diameter and the particle size distribution were obtained from the analysis of the autocorrelation functions using the cumulants method. At least three measurements at 25 °C were made for each sample (0.10% *w/w* dispersions) with an equilibrium time of 2 min before starting the measurement.

### 2.3. Synthesis and Characterization of mPEG_1k_-BPA

**Scheme 3:**
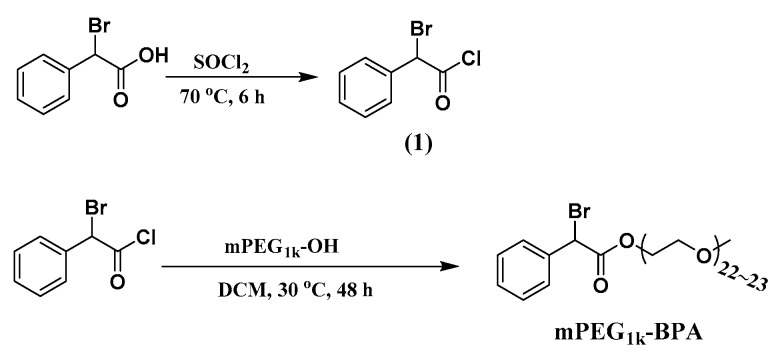
The synthetic route of mPEG_1k_-BPA.

The polyethylene glycol monomethyl ether 2-bromo-2-phenylacetate (mPEG_1k_-BPA) was synthesized according to the method in the reference [30]. The typical procedure is shown in Scheme 3. Firstly, 20 mL of dichlorosulfane and 8.6 g of 2-bromo-2-phenylacetic acid (40 mmol) were added to a 50 mL three-necked flask. The temperature was raised to 70 °C under magnetic stirring and refluxed for 6 h. After cooling to room temperature, the remaining dichlorosulfane was removed by rotary evaporation to obtain 2-bromo-2-phenylacetyl chloride (1). Its structure was clearly characterized by NMR spectroscopy (^1^H-NMR (300 MHz, CDCl_3_, TMS, δ, ppm): 7.59–7.32 (m, 5H), 5.94 (s, 1H).

Then, in a 250 mL three-necked flask, 8.1 g of mPEG_1k_, 0.5 mL of triethylamine and 150 mL of anhydrous dichloromethane were added. 5 g of (1) was added dropwise with a constant pressure dropping funnel under ice bath (0 °C). After the addition was completed, increasing temperature to 30 °C for 36 h. The dichloromethane was removed by rotary evaporation. The product was dissolved with a small amount of tetrahydrofuran (THF) and then precipitated in anhydrous ether. This process was repeated three times. Then a white solid (mPEG_1k_-BPA) was collected by suction filtration. Its structure was verified by NMR spectroscopy (^1^H-NMR, 300 MHz, CDCl_3_, TMS, δ, ppm): 7.60–7.31 (m, 5H), 5.39 (s, 1H), 4.34 (d, 2H), 3.98–3.51(m, 90H), 3.38 (s, 3H), 2.10 (s, 2H).

### 2.4. General Procedure for the Polymerization of BnMA or HPMA

We conducted the polymerization in ampules under an argon (Ar) atmosphere, and the light source is blue light-emitting diode (LED) light strip (λ_max_ = 464 nm, 0.15 mW/cm^2^). A typical polymerization procedure for the molar ratio of [BnMA]_0_/[mPEG_1k_-BPA]_0_/[NaI]_0_/[TEA]_0_ = 20/1/2/0.5 is shown as follows: a mixture of mPEG_1k_-BPA (14.8 mg, 0.015 mmol), NaI (4.4 mg, 0.03 mmol), BnMA (50 μL, 0.30 mmol), TEA (1.0 μL, 0.0075 mmol), and methanol (0.50 mL) was added to a dried ampule (2 mL) with a stir bar. The mixed solution was a pale yellow homogeneous solution. After three freeze-pump-thaw cycles to eliminate the dissolved oxygen and provide an Ar atmosphere, the ampule was flame-sealed. The mixture was transferred to a stirring device equipped with a blue LED strip, cooling with an electric fan to remove heat from the LED strip. The polymerization was maintained at room temperature (25 °C). After a desired polymerization time, the ampule was moved to a dark place, and 20 μL of the polymer solution was pipetted into a solvent of DMSO-*d*_6_ and subjected to ^1^H-NMR characterization for monomer conversion. For the polymerization of HPMA, a mixture of polymerization solution was obtained with the molar ratio of [HPMA]_0_/[mPEG_1k_-BPA]_0_/[NaI]_0_/[TEA]_0_ = 300/1/2/1 (PEG1k-BPA: 14.8 mg, 0.015 mmol; NaI: 4.4 mg, 0.03 mmol; HPMA: 0.5 mL, 3.5 mmol; TEA: 1.72 μL, 0.012 mmol) in a mixed solvent of 1.67 mL of methanol and 0.83 mL of water, and the rest procedure was the same as that for the polymerization of BnMA mentioned above.

## 3. Results and Discussion

### 3.1. Polymerization Mechanism

Firstly, blue light plays a vital role in the photo-BIT-RDRP system. Our previous research demonstrated that the type of light source has a significant effect on the monomer conversion, molecular weight, and molecular weight distribution of the resultant polymers [27]. Compared with light sources of other wavelengths, the blue light we selected has relatively good control over the polymerization process. On the other hand, the occurrence and stop of the polymerization reaction strictly follow the light on and off, which makes the polymerization method have excellent spatiotemporal controllability [27,29].

**Figure 1:**
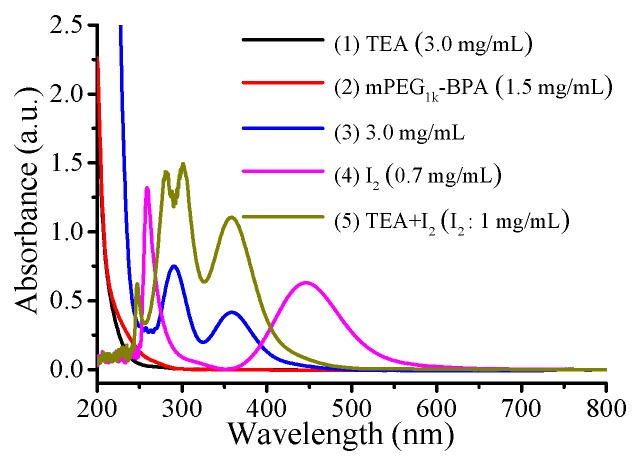
UV-vis absorption spectra of the (1) TEA, (2) mPEG_1k_-BPA, (3) polymerization conditions: [BnMA]_0_/[mPEG_1k_-BPA]_0_/[NaI]_0_/[TEA]_0_ = 20/1/2/0.5, *V*_BnMA_ = 50 μL, under irradiation with blue LED light (λ_max_ = 464 nm, 0.15 mW cm^−2^) at room temperature (25 °C) after 7 h, (4) I_2_, (5) TEA/I_2_ = 4:1. The samples were diluted with methanol before measurement.

**Scheme 4:**
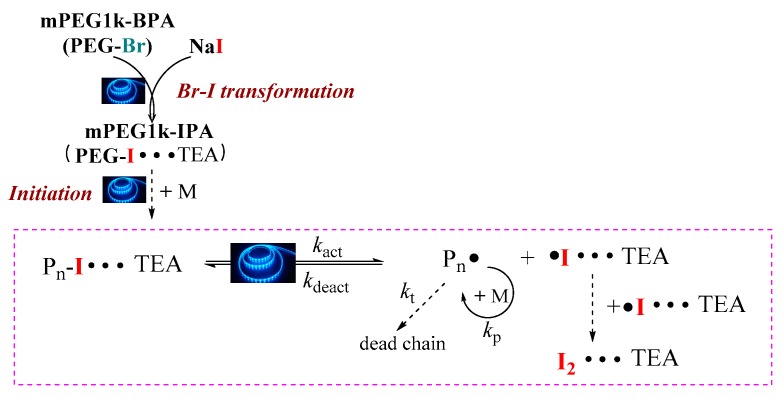
Proposed polymerization mechanism by using one-step photo-BIT-RDRP strategy.

As reported by our previous work, the photo-BIT-RDRP process will result in the formation of a small amount of iodine [27]. Therefore, the UV-vis absorption spectra were used to verify whether I_2_ was generated during the polymerization. We measured the UV-visible absorption spectra of TEA, mPEG_1k_-BPA and the reaction solution after 7 h of polymerization ([BnMA]_0_/[mPEG_1k_-BPA]_0_/[NaI]_0_/[TEA]_0_ = 20/1/2/0.5) (Figure 1). TEA and mPEG_1k_-BPA have absorption in the wavelength range of 200–300 nm, and no absorption in the wavelength range of more than 300 nm. However, after 7 h of polymerization, absorption peaks appeared at 365 nm and 297 nm. In comparison to the UV-vis absorption spectra of the I_2_ and I_2_-TEA complexes, there actually exists the absorption peak of the resulting I_2_-TEA complex. This proves that there is indeed a small amount of I_2_ formation during the polymerization. On the other hand, the polymerization solution was pale yellow before irradiation with blue LED light. However, after irradiation, the color of polymerization solution gradually became darker, which proved the formation of a small amount of iodine again. Therefore, we can propose the polymerization mechanism of this one-step photo-BIT-RDRP, which is similar with that of typical reversible complexation mediated polymerization (RCMP) as reported previously [31,32,33,34], and is shown in Scheme 4. The ATRP macroinitiator mPEG1k-BPA (PEG-Br) was first transferred into mPEG_1k_-IPA (PEG-I) in the presence of NaI under irradiation with blue LED light. The in situ generated PEG-I was used as the starting RCMP macroinitiator to initiate the monomer polymerization and therefore to form the propagating polymer chains P_n_-I. It should be noted that the halogen bond interaction can be formed between TEA and iodine in the presence of catalyst TEA [17,35]. Thereby the C-I bond can be cleaved easily under irradiation with blue LED light to generate the carbon-centered propagating radicals (P_n_•) and iodine radical/H_2_O complex (I•/H_2_O complex). At the same time P_n_• can reversibly combine the iodine radical to form a dormant species P_n_-I, and a small amount of the free iodine radicals can also combine to form I_2_ during the polymerization process as observed in Figure 1.

### 3.2. In Situ Photo-BIT-RDRP of BnMA and Its Self-Assembly Behavior

**Figure 2:**
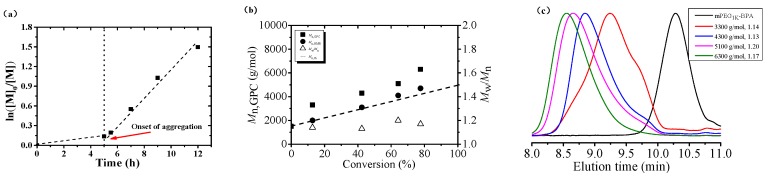
ln([M]_0_/[M]) as a function of time (**a**), number-average molecular weight (*M*_n,GPC_) and molecular weight distribution (*M*_w_/*M*_n_) versus monomer conversion (**b**) and GPC elution curves (**c**) for photo-BIT-RDRPof BnMA. Polymerization conditions: [BnMA]_0_/[mPEG_1k_-BPA]_0_/[NaI]_0_/[TEA]_0_ = 20/1/2/0.5, *V*_BnMA_ = 50 μL, *V*_methanol_ = 0.5 mL, under irradiation with blue LED light (*λ*_max_ = 464 nm, 0.15 mW/cm^2^) at room temperature (25 °C); BnMA concentration at 20.0 wt %.

**Figure 3:**
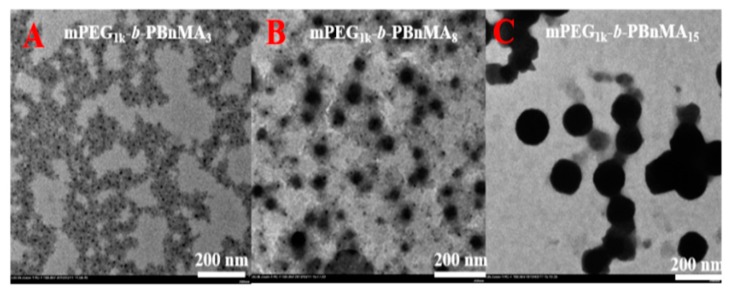
TEM images (**A**–**C**) of micelles from mPEG_1k_-*b*-PBnMA_x_ (x = 3, 8, and 15, respectively) via one-step photo-BIT-RDRP-PISA using BnMA as the monomer. The samples obtained by the BnMA concentration at 20.0 wt %.

We investigated the polymerization behaviors of BnMA using mPEG_1k_-BPA as the macroinitiator under irradiation with blue LED light at room temperature. As can be seen from Figure 2a, ln([M]_0_/[M]) grows very slowly over time in 0 to 5 h, indicating that the increase in the polymerization degree of PBnMA before 5 h is not enough to achieve micelle nucleation. However, after 5 h, ln([M]_0_/[M]) increases linearly with polymerization time. It is due to the increase in viscosity after micelle nucleation, and the monomer is encapsulated inside mPEG_1k_-*b*-PBnMA, resulting in an increase in local monomer concentration. Therefore, the polymerization rate is significantly accelerated. From Figure 2b, the molecular weights of the resultant amphiphilic block copolymers increase linearly with monomer conversion, and the molecular weight distributions keep narrow (*M*_w_/*M*_n_ < 1.20). It is noted that there are some deviations in molecular weights measured by GPC (*M*_n,GPC_) from the theoretical ones (*M*_n,th_) since PMMA was used as the standard for calibration in GPC; however, the molecular weights determined by NMR spectra (*M*_n,NMR_) was close to their corresponding *M*_n,th_ values. In addition, as shown in Figure 2c, there is an obvious peak shift after block polymerization with BnMA. These facts indicate that the photo-BIT-RDRP of BnMA is consistent with the typical feature of RDRP. The self-assembly morphology of the mPEG_1k_-*b*-PBnMA_x_ (x = 3, 8, and 15) at different degrees of polymerization is shown in Figure 3 (the detailed information is shown in Table 1). Figure 3A is a topographical view of the block PBnMA with the degree of polymerization (DP) of 3. At this time, the insoluble segments are shorter, so that the particle size of the micelles formed is small (about 12.8 nm). In Figure 3B, for DP 8, the particle size increases to 43.2 nm. As shown in Figure 3C, the particle size of the assembled spherical polymer micelles increased to 177.1 nm correspondingly.

### 3.3. In Situ Photo-BIT-RDRP of HPMA and Its Self-Assembly Behavior

**Figure 4:**
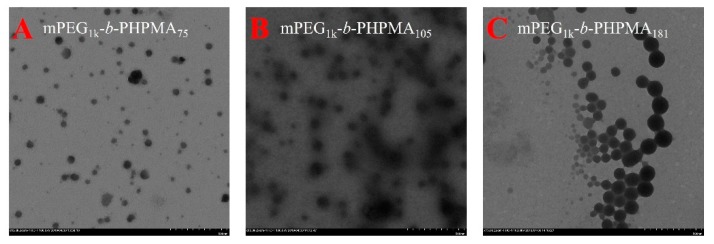
TEM images (**A**–**C**) of micelles from mPEG_1k_-*b*-PHPMA_x_ (x = 75, 105, and 181, respectively) via one-step photo-BIT-RDRP-PISA using HPMA as the monomer. The samples obtained by the HPMA concentration at 20.0 wt %.

HPMA has considerable solubility in methanol or water, while PHPMA has poor solubility, especially for high molecular weight PHPMA in water. Therefore, HPMA is also an ideal monomer for a PISA process. We chose a mixed solution of methanol and water as the solvent. Figure 4 is a topographical view of the assembly of mPEG_1k_-*b*-PHPMA_x_ (x = 75, 105, 181) with different degrees of polymerization observed by TEM (polymerization information is shown in Table 2). When the DP is 75, the particle size of the self-assembled nanoparticles is about 34.2 nm. Increasing DP to 105, the particle size of the nanoparticles increased to 50.1 nm. Further increasing the DP to 181, the nanoparticles correspondingly increased to 102.9 nm. Therefore, the size of the resultant nanoparticles can be easily controlled by adjusting the degree of polymerization during the photo-BIT-RDRP-PISA process.

## 4. Conclusions

In summary, we have synthesized a water-soluble macroinitiator mPEG_1k_-BPA and realized a one-step in situ photo-BIT-RDRP-PISA process under irradiation with blue LED light at room temperature, successfully obtaining mPEG_1k_-*b*-PBnMA and mPEG_1k_-*b*-PHPMA amphiphilic block copolymer micelles in situ. This strategy effectively improves the problem of the active chain end (C-I) loss caused by two-step bromine-iodine transformation RDRP-PISA process, and also greatly simplifies the synthesis step, which provides a promising method for the synthesis of polymeric nanoparticles by photo-BIT-RDRP-PISA strategy.

## Figures and Tables

**Table 1:** Polymerization of BnMA via photo-BIT-RDRP.

Entry	Time(h)	Conv. *^a^* (%)	*M*_n,th_*^b^* (g/mol)	*M*_n,NMR_*^c^* (g/mol)	*M*_n,GPC_ (g/mol)	*M*_w_/*M*_n_	DP *^d^*	Particle Size (nm) *^e^*
A	5.5	12.8	2000	2000	3300	1.14	3	12.8 ± 2.4
B	7	42.4	3000	3100	4300	1.13	8	43.2 ± 2.0
C	12	77.6	4200	4700	6300	1.17	15	177.1 ± 2.7

Polymerization conditions: [BnMA]_0_/[mPEG_1k_-BPA]_0_/[NaI]_0_/[TEA]_0_ = 20/1/2/0.5, *V*_BnMA_ = 50 μL, *V*_methanol_ = 0.5 mL, under irradiation with blue LED light (*λ*_max_ = 464 nm, 0.15 mW cm^−2^) at room temperature (25 °C). *^a^* Monomer conversion was calculated from ^1^H NMR spectra results. *^b^ M*_n,th_ = [BnMA]_0_/[mPEG_1k_-BPA]_0_ × *M*_BnMA_ × Conv.% + *M*_n,mPEG__1k__-BPA_. *^c^* Molecular weight calculated from ^1^H NMR spectra results. *^d^* Degree of polymerization calculated from Conv.%. *^e^* Obtained from TEM images.

**Table 2:** Polymerization of HPMA via photo-BIT-RDRP.

Entry	Time (h)	Conv. *^a^* (%)	*M*_n,th_*^b^* (g/mol)	*M*_n,GPC_ (g/mol)	*M*_w_/*M*_n_	DP *^c^*	Particle Size (Ð) *^d^*
A	6	25	12,300	17,200	1.34	75	34.2 (0.208)
B	8	35.1	16,700	22,300	1.28	105	50.1 (0.163)
C	12	60.4	27,600	33,500	1.28	181	102.9 (0.260)

Polymerization conditions: [HPMA]_0_/[mPEG_1k_-BPA]_0_/[NaI]_0_/[TEA]_0_ = 300/1/2/1, *V*_HPMA_ = 0.5 mL, *V*_methanol_ = 1.67 mL, *V*_water_ = 0.83 mL, under irradiation with blue LED light (*λ*_max_ = 464 nm, 0.15 mW cm^−2^) at room temperature (25 °C). *^a^* Monomer conversion was calculated from ^1^H NMR spectra results. *^b^ M*_n,th_ = [HPMA]_0_/[mPEG_1k_-BPA]_0_ × *M*_HPMA_ × Conv.% + *M*_n,mPEG__1k__-BPA_. *^c^* Degree of polymerization calculated from Conv.%. *^d^* Obtained by DLS.
